# Pulmonary infection caused by *Mycobacterium kansasii*: findings on computed tomography of the chest*

**DOI:** 10.1590/0100-3984.2015.0078

**Published:** 2016

**Authors:** Roberto Mogami, Telma Goldenberg, Patricia Gomes Cytrangulo de Marca, Fernanda Carvalho de Queiroz Mello, Agnaldo José Lopes

**Affiliations:** 1 PhD, Adjunct Professor of Radiology at the Universidade do Estado do Rio de Janeiro (UERJ), Head of the Department of Radiology at the Hospital Universitário Pedro Ernesto (HUPE), Rio de Janeiro, RJ, Brazil.; 2 Master's Student in the Graduate Program in Clinical Medicine at the Universidade Federal do Rio de Janeiro (UFRJ), Physician at the Centro de Referência Professor Hélio Fraga (CRPHF) of the Escola Nacional de Saúde Pública Sergio Arouca / Fundação Oswaldo Cruz (ENSP/Fiocruz), Rio de Janeiro, RJ, Brazil.; 3 Graduate Student in Radiology at the Universidade do Estado do Rio de Janeiro (UERJ), Rio de Janeiro, RJ, Brazil.; 4 PhD, Director of the Instituto de Doenças do Tórax, Associate Professor of Pulmonology at the Faculdade de Medicina da Universidade Federal do Rio de Janeiro (UFRJ), Rio de Janeiro, RJ, Brazil.; 5 PhD, Adjunct Professor of Pulmonology at the Universidade do Estado do Rio de Janeiro (UERJ), Rio de Janeiro, RJ, Brazil.

**Keywords:** Mycobacterium infections, nontuberculous, Tomography, X-ray computed, Lung/pathology

## Abstract

**Objective:**

To describe the main tomography findings in patients diagnosed with pulmonary infection caused by *Mycobacterium kansasii*.

**Materials and Methods:**

Retrospective study of computed tomography scans of 19 patients with pulmonary infection by *M. kansasii*.

**Results:**

Of the 19 patients evaluated, 10 (52.6%) were male and 9 (47.4%) were female. The mean age of the patients was 58 years (range, 33-76 years). Computed tomography findings were as follows: architectural distortion, in 17 patients (89.5%); reticular opacities and bronchiectasis, in 16 (84.2%); cavities, in 14 (73.7%); centrilobular nodules, in 13 (68.4%); small consolidations, in 10 (52.6%); atelectasis and large consolidations, in 9 (47.4%); subpleural blebs and emphysema, in 6 (31.6%); and adenopathy, in 1 (5.3%).

**Conclusion:**

There was a predominance of cavities, as well as of involvement of the small and large airways. The airway disease was characterized by bronchiectasis and bronchiolitis presenting as centrilobular nodules.

## Abstract (pt)

**Objetivo:**

Descrever os achados tomográficos de pacientes com diagnóstico de infecção pulmonar pelo *Mycobacterium kansasii*.

**Materiais e Métodos:**

Estudo retrospectivo dos exames de tomografia computadorizada do tórax de 19 pacientes com infecção pulmonar pelo *M. kansasii*.

**Resultados:**

Dos 19 pacientes avaliados, 10 (52,6%) eram do sexo masculino e 9 (47,4%) eram do sexo feminino. A média de idade do grupo foi 58 anos, com variação entre 33 e 76 anos. As alterações encontradas nos exames de tomografia computadorizada foram distorção arquitetural em 17 pacientes (89,5%), opacidades reticulares e bronquiectasias em 16 (84,2%), cavidades em 14 (73,7%), nódulos centrolobulares em 13 (68,4%), pequenas consolidações em 10 (52,6%), atelectasias e grandes consolidações em 9 (47,4%), bolhas subpleurais e enfisema em 6 (31,6%) e linfonodomegalias em 1 paciente (5,3%).

**Conclusão:**

Houve predomínio de cavidades e do padrão de acometimento de pequenas e grandes vias aéreas. A doença de vias aéreas foi caracterizada por bronquiectasias e bronquiolites que se manifestaram como nódulos centrolobulares.

## INTRODUCTION

Nontuberculous mycobacteria (NTM) are organisms that are ubiquitous in nature and can cause infections in immunocompetent patients. They can be divided into two groups, according to the growth pattern: slow and fast. Slow-growing NTM include *Mycobacterium avium, Mycobacterium intracellulare* (*Mycobacterium avium-intracellulare* complex) and *Mycobacterium kansasii*, whereas fast-growing NTM include *Mycobacterium abscessus, Mycobacterium fortuitum* and *Mycobacterium chelonae*^(1)^.

NTM can be isolated in water, soil, milk, and animal flesh. The infection can be acquired by inhalation, ingestion, or direct inoculation. In the case of infection with *M. kansasii*, the main reservoir is in tap water and the contamination is via the airway^(2)^. The disease has a chronic, indolent course, and diagnosis is difficult because isolation of the agent in bronchoalveolar lavage fluid may represent only colonization of the airway^(3)^. The type of NTM most often isolated is *M. avium-intracellulare* complex^(4)^, followed by *M. kansasii*^(2,5)^.

Initially, two patterns of pulmonary involvement by NTM were identified: cavitary disease in the upper lobes; and nodular disease with bronchiectasis, mainly in the middle lobe and lingula^(6)^. The first pattern, which was reported most frequently in the 1980s, mimicked the classic aspect of tuberculosis. Prior to the advent of computed tomography (CT), authors such as Zvetina et al.^(7)^ wrongly attempted to establish radiographic differences between the cavities caused by *M. kansasii* and those caused by *M. tuberculosis*. The typical patient showing the cavitary disease pattern has some type of comorbidity, such as alcoholism, cancer, chronic obstructive pulmonary disease, cystic fibrosis, bronchiectasis, and pulmonary fibrosis^(8)^. The second pattern occurs predominantly in nonsmoking middle-aged women who have chronic cough and expectoration. In this subtype, conditions that predispose to NTM are uncommon. The disease is known by some as Lady Windermere syndrome^(9,10)^. In a review of the CT scans of 22 patients with NTM, Jeong et al.^(11)^ found the pattern of nodular disease with bronchiectasis to be the most common. The corresponding histopathology finding was bronchiolectasis with bronchial and peribronchial inflammatory infiltrates, with or without granuloma formation. Pulmonary involvement with bronchiectasis and signs of bronchiolitis is not specific to a given strain of mycobacteria^(4,11)^.

In addition to the two patterns mentioned above, Erasmus et al.^(3)^ divided patients infected with NTM into three specific categories, on the basis of clinical and radiological changes: asymptomatic patients with nodules; patients with achalasia and thoracic abnormalities; and immunocompromised patients with thoracic changes. In the last category, the radiological pattern is slightly different^(12)^, the most common findings being interstitial involvement, in 51.3%; consolidations, in 37.5%; pleural effusion, in 36.3%; and adenopathy, in 31.3%.Matveychuk et al.^(5)^ found differences among the various types of NTM infection in terms of the radiographic findings. The presence of cavities was more common in infections caused by *M. kansasii*, as were lesions in the upper lobes and unilateral lung involvement. Those authors also reported that pleural effusion and mediastinal adenopathy were found in few of the patients infected with *M. kansasii*^(5)^. Similarly, Shitrit et al.^(2)^ found that, among patients infected with *M. kansasii*, the predominant aspects were cavitary disease (in 54%) and major involvement of the upper lobes (in 82%). In both of those studies, the authors observed no pleural effusion or adenopathy in any of the patients^(2,5)^. However, Matveychuk et al.^(5)^ and Shitrit et al.^(2)^ both used routine chest X-ray alone to identify abnormalities in that patient population.

Given that chest CT differs from conventional chest X-ray, in terms of sensitivity and specificity, with better spatial resolution and greater discrimination of densities^(13,14)^, it is essential to evaluate its role in the diagnosis of thoracic abnormalities in patients infected with *M. kansasii*. To our knowledge, there have been no studies aimed at discriminating the CT findings in such patients in Brazil. Because of the difficulty in establishing the diagnosis of NTM lung infections, the aim of this study was to describe the frequency of abnormalities found on CT scans of the chest in a group of patients with pulmonary infection caused by *M. kansasii*.

## MATERIALS AND METHODS

We evaluated the chest CT scans of 19 patients with proven pulmonary infection caused by *M. kansasii*, treated at our institution between 2006 and 2014. The diagnosis of pulmonary infection with *M. kansasii* (rather than colonization alone) was made by combining clinical, radiological, and microbiological criteria, as recommended by the American Thoracic Society^(15)^. Microbiological confirmation was obtained by applying the following combinations of criteria: three positive cultures with negative smear microscopy results or two positive cultures and one positive sputum smear microscopy; one positive smear microscopy and one positive culture or just one positive culture of bronchoalveolar lavage fluid; one positive culture or one pathology examination showing inflammatory granuloma formation, with or without a smear-positive lung biopsy^(16)^. CT scans, performed in various scanners, produced documentation of sequential slices in a parenchymal window (including highresolution acquisition) and a mediastinal window, without administration of intravenous contrast. The CT scans were interpreted separately and randomly by a thoracic radiologist with more than 10 years of experience in the specialty. The abnormalities were defined and classified in accordance with the criteria established by the Fleischner Society^(17)^ and the Brazilian Illustrated Consensus terminology of descriptors and fundamental patterns on chest CT scans^(18)^. In addition, for the purposes of interpretation, consolidations were divided into large and small, a cut-off value of 3 cm being adopted to differentiate between the two.

## RESULTS

Among the 19 patients evaluated, the mean age was 58 years (range, 33-76 years), 10 (52.6%) were male, and 9 (47.4%) were female.

**Figure 1:**
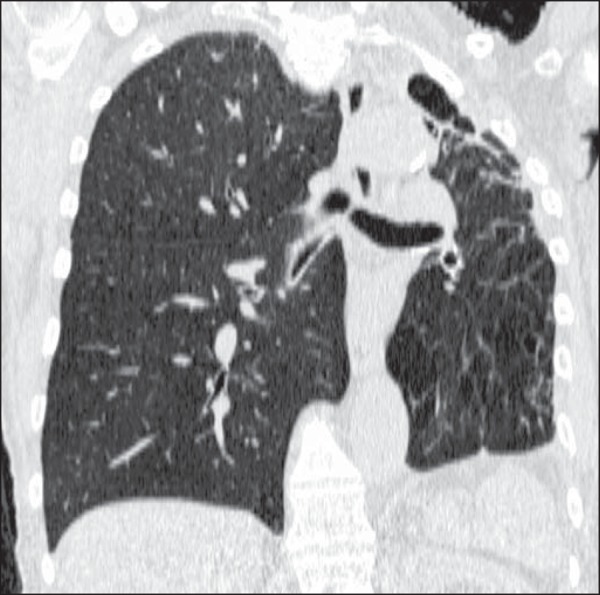
Left lung volume loss, with architectural distortion and reticular opacities.

**Figure 2:**
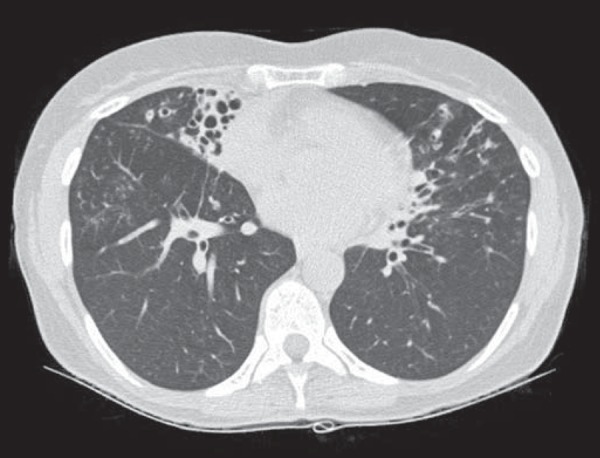
Bronchiectasis and nodules in the middle lobe, lower right lobe, and lingula. In the middle lobe, atelectasis and architectural distortion caused by destruction of the lung parenchyma can be seen.

**Figure 3:**
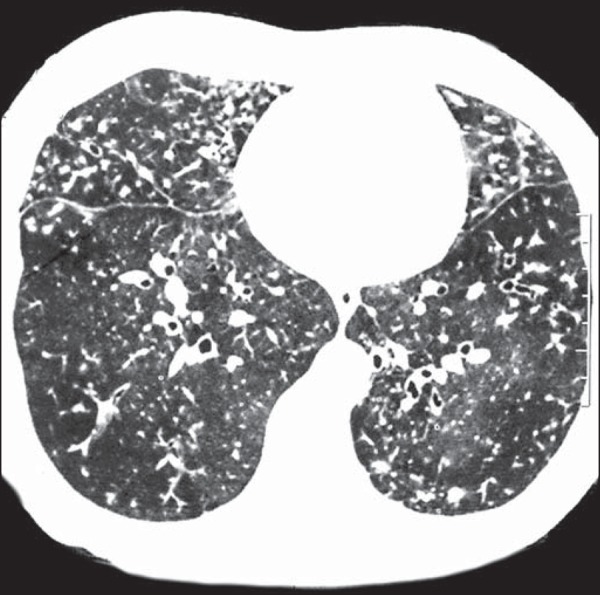
Areas showing a mosaic pattern of attenuation and tree-in-bud opacities.

**Figure 4:**
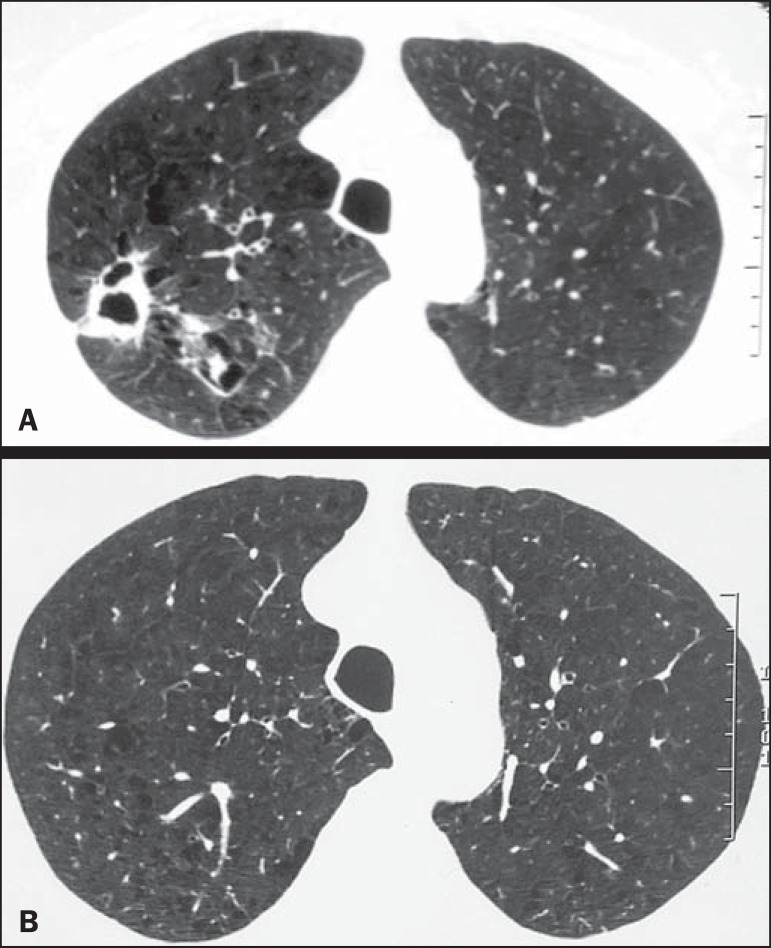
**A:** Cavity in the right upper lobe, accompanied by signs of centrilobular emphysema. **B:** After successful treatment, the cavity disappeared.

**Figure 5:**
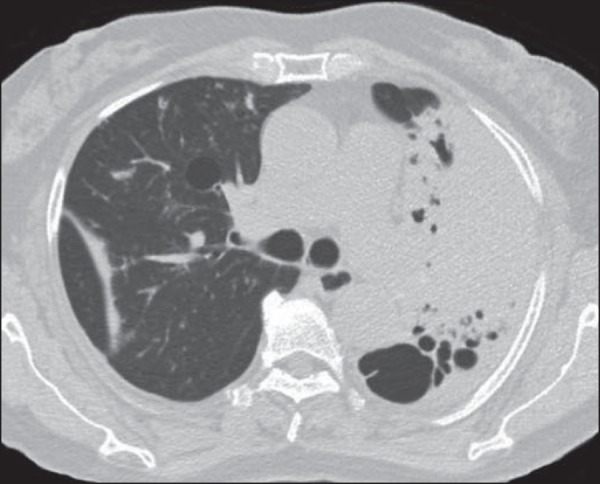
Consolidation and cavity in the left lung.

**Figure 6:**
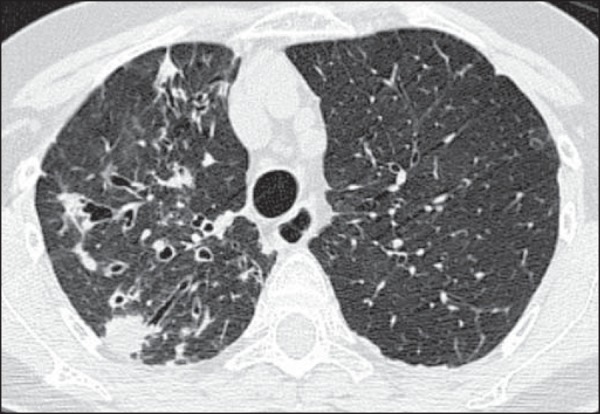
Subsegmental consolidations and bronchiectasis.

The main changes seen on the 19 CT scans evaluated were architectural distortion, in 17 patients (89.5%), as shown in Figure 1; reticular opacities, in 16 (84.2%), also shown in Figure 1; bronchiectasis, in 16 (84.2%), as shown in Figures 2 and 3; cavities, in 14 (73.7%), as shown in Figures 4 and 5; and centrilobular nodules, in 13 (68.4%), as shown in Figures 2 and 3. Other changes were small (≤ 3 cm) consolidations, in 10 cases (52.6%), as shown in Figure 6; atelectasis, in 9 (47.4%), as shown in Figure 1; large (> 3 cm) consolidations, also in 9 (47.4%), as shown in Figure 5; subpleural blebs and emphysema, in 6 (31.6%), as shown in Figure 4; and adenopathy, in 1 (5.3%).

In 10 (52.6%) of the 19 cases, bronchiectasis was observed in the upper lobes, whereas it was observed in the lower lobes in 6 cases (31.6%) and in the middle lobe/lingula in 3 (15.8%). One patient (5.3%) had calcified nodules and the remaining 18 (94.7%) had centrilobular nodules attributed to filling of the bronchioles (infectious bronchiolitis).

Of the 19 patients, 12 (85.7%) showed cavities in the upper lobes, only two patients (14.3%) showing cavities in the lower lobes. No cavities were observed in the middle lobe or lingula.

## DISCUSSION

The assessment of pulmonary infections by CT has been the subject of a number of recent publications in the radiology literature of Brazil^(19-24)^. In the present study, the evaluation of the changes found on CT scans showed that the main findings, in order of frequency, were architectural distortion, changes in the interstitial space, cavities, and centrilobular nodules. It is known that there are two main patterns of involvement by NTM: cavities in the upper lobes (similar to tuberculosis) and bronchiectasis/bronchiolitis^(6,7,9,10)^. In our sample, those two patterns of involvement occurred at similar frequencies, different than in the study conducted by Takahashi et al.^(25)^, who, in a sample of 29 patients infected with *M. kansasii* and evaluated by CT, identified cavity lesions in 83% of cases and bronchiectasis in only 27.6%. It is possible that the differences in the frequencies of comorbidities and rates of tuberculosis prevalence between the populations evaluated explain, at least in part, the discrepancies in the results of the two studies.

Some authors refer to the pattern of preferential involvement of the airways, when bronchiectasis occurs in the middle lobe/lingula and is associated with NTM, as Lady Windermere syndrome^(9,10)^. This syndrome was first described in 1992 by Reich et al.^(26)^, who associated it with the clinical features inspired by the personality of the protagonist of the Oscar Wilde play "Lady Windermere's Fan", who concealed her cough for social reasons (shame in coughing and expectorating). In Lady Windermere syndrome, in addition to multiple areas of bronchiectasis, there is radiological evidence of centrilobular nodules and a tree-in-bud pattern, together with voluntary cough suppression, leading to a nonspecific inflammatory process, at a site where drainage is difficult, which could explain its pathogenesis^(27,28)^. In our sample, however, bronchiectasis was most prevalent in the upper lobes (52.6%). That differs from the distribution reported in the literature for individuals infected with *M. kansasii* or *M. avium-intracellulare* complex^(5,6)^.

The high frequency of architectural distortion seen on the CT scans evaluated in the present study might be due to the NTM itself or secondary to other comorbidities that can lead to fibrosis, such as tuberculosis. CT can identify architectural changes that, on conventional X-rays, can be confused with bronchiectasis and areas of fibrosis. In addition, CT clearly demonstrates the important features of the cavities, such as the borders, the wall thickness, and the contents. In the present study, cavitary lesions in the upper lobes predominated, being observed in 85.7% of the cases, similar to what has been reported by other authors^(26)^.

On the CT scans, we observed large and small consolidations in high proportions of the patients (47.4% and 52.6%, respectively). Large consolidations were defined as those > 3 cm and could represent injuries that converged and expanded to broader regions of the lung parenchyma. In the follow-up of the patients with such consolidations, we observed that, in some cases, the confluence of those consolidations worsened the tomographic alterations^(3)^.

Similar to what was reported in the study conducted by Takahashi et al.^(25)^, we observed a low incidence of adenopathy and pleural lesions. This is important information in Brazil, where the incidence of tuberculosis is very high. That is because a finding of pleural involvement or lymph node enlargement makes a diagnosis of NTM less likely, and when there is concomitant suspicion of tuberculosis, the second condition-association of this two diseases-is more plausible.

Although chronic obstructive pulmonary disease is a predisposing factor for infection with NTM, we observed a low prevalence of emphysematous lesions and pulmonary blebs (31.6% for both) on CT scans. For the most part, the literature does not mention these findings in radiological examinations of infection with *M. kansasii*^(26)^. In a sample of patients with pulmonary infection caused by *M. avium-intracellulare* complex, Song et al.^(29)^ also reported a low prevalence of blebs and emphysema (11% and 32%, respectively).

A critical analysis of the results of this study and its limitations is relevant. First, because this was a retrospective study based on surveys conducted at different institutions, the documentation did not always refer to images obtained during an expiratory breath hold, which would be important to assess air trapping and consequently (indirectly) bronchiolitis. Second, the CT findings were reviewed by only one radiologist, which makes them susceptible to bias. Third, we did not determine whether the tomographic findings correlated with the clinical findings and with pulmonary function. Despite these limitations, we believe our results make an important contribution, given that there have been few studies evaluating tomographic findings in this group of patients. Future studies, using a statistical analysis involving a larger sample, a control group, and longitudinal follow-up of these patients, will confirm our observations.

In conclusion, the predominant features in our sample of 19 patients with pulmonary infection caused by *M. kansasii* were cavities and airway involvement (characterized by bronchiectasis and changes attributed to the filling of the bronchioles).
